# A bibliometric analysis of international publication trends in brain atrophy research (2008–2023)

**DOI:** 10.3389/fneur.2024.1348778

**Published:** 2024-01-31

**Authors:** Juwei Wang, Tingting Chen, Jiayi Xie, Sheng Zhao, Yue Jiang, Huihe Zhang, Wenzong Zhu

**Affiliations:** ^1^Zhejiang Chinese Medical University, Department of Graduate College, Hangzhou, China; ^2^Department of Acupuncture, First Affiliated Hospital of Wenzhou Medical University, Wenzhou, China; ^3^Department of Neurology, Wenzhou Hospital of Traditional Chinese Medicine, Wenzhou, China; ^4^Department of Neurology, Wenzhou Hospital of Integrated Traditional Chinese and Western Medicine, Zhejiang Chinese Medical University, Wenzhou, China

**Keywords:** CiteSpace, VOSviewer, bibliometric, visual perception, brain atrophy

## Abstract

**Background:**

Brain atrophy is a type of neurological and psychiatric disorder characterized by a decrease in brain tissue volume and weight for various reasons and can have a serious impact on the quality of life of patients. Although there are many studies on brain atrophy, there is a lack of relevant bibliometric studies. Therefore, this study aims to provide a visual analysis of global trends in brain atrophy research over the past 16 years.

**Methods:**

CiteSpace and VOSviewer were used to visually analyze publication output, scientific collaborations, cocitations, publishing journals, and keywords to determine the current status and future trends of brain atrophy research. Materials published from 2008 to 2023 were collected from the Web of Science Core Collection (WoSCC) database. This study placed no restrictions on the types of literature and focused on English language publications.

**Results:**

A total of 3,371 publications were included in the analysis. From 2008 to 2023, the number of publications increased annually. In terms of national and academic institutions, universities in the United States and University College London rank first in publication out. Barkhof Frederik and Zivadinov Robert are the most prolific researchers in this field. The publication with the highest cocitation strength is “Deep gray matter volume loss drives disability worsening in multiple sclerosis.” Keyword clustering analysis showed that “Alzheimer’s disease” and “multiple sclerosis” are current popular topics. The analysis of emergent words indicates that “cerebral small vessel disease,” “neurodegeneration,” and “cortex/gray matter volume” may become hot research topics in the coming years.

**Conclusion:**

This study analyses papers on brain atrophy from the past 16 years, providing a new perspective for research in this field. In the past 16 years, research on brain atrophy has received increasing attention. The quality of articles in this field is generally high. Extensive national cooperation already exists. The statistical results indicate that a stable core author group in the field of brain atrophy has almost formed.

## Introduction

Brain atrophy is a type of neurological and psychiatric disorder characterized by a decrease in brain tissue volume and weight that is commonly seen in the elderly but also can occur in adolescents and young children. Imaging of these patients usually shows widening and deepening of the sulci, flattening of the gyri, and enlargement of the ventricles, cisterns, and subarachnoid space. There are many causes of cerebral atrophy, including but not limited to Alzheimer’s disease, Multiple sclerosis, aging, cerebrovascular diseases, epilepsy, brain tumors, and encephalitis. In addition, other studies have reported that the risk factors of the development of brain atrophy include diabetes, hypertension, heart disease, and alcohol consumption, and the reduction in brain volume usually accelerates with age (1).

The symptoms of cerebral atrophy include cognitive impairment (e.g., memory decline, intellectual impairment), personality changes (e.g., depression, paranoia), neurological symptoms (e.g., dizziness, headaches), and degradation of limb motor function. Among these symptoms, cognitive impairment is the most significant change. Although patients with brain atrophy may not have any symptoms initially, their risk of developing dementia increases (2). Currently, the treatment for brain atrophy is mainly symptomatic and primary treatment, but brain atrophy cannot be completely cured. In addition, brain atrophy spans various diseases, such as Alzheimer’s disease, multiple sclerosis, Parkinson’s disease, and other well-known related diseases (3–5). The extensive volume of literature also poses a challenge for comprehensive reviews, making it cumbersome for researchers to efficiently identify key information in the field. Critical insights, such as leading countries, institutions, or authors from a statistical perspective, become challenging to discern amidst the vast amount of available material.

Bibliometrics is the study of academic publications (papers, books, conferences, etc.) in a certain field, using statistical data analysis to determine the current research status, hot topics, and future trends (6). This scientific analysis can be completed with specialized software, with CiteSpace and VOSviewer being the most widely used (7). CiteSpace was developed by Professor Chaomei Chen from Drexel University in the United States (8). VOSviewer is published by Nees Jan van Eck and Ludo Walterman from Leiden University in the Netherlands (9). This article employs VOSViewer as a supplemental tool and mainly relies on CiteSpace; by selecting appropriate literature, analyzing the frequency, journals, countries, institutions, and keywords of brain atrophy, and summarizing the research status and development, this study provides a reference for future research.

## Materials and methods

### Data sources and retrieval strategies

The materials were collected from the Web of Science Core Collection (WoSCC) database. Compared to Scopus, EI Compendex, CSSCI and CNKI, the WoSCC database is more comprehensive and reliable. We used the following search terms: TS = (“Brain Atrophy” OR “Cerebral Atrophy” OR “Encephal atrophy” OR “Gray Matter Atrophy” OR “Subcortical Atrophy”). The search was completed on October 9, 2023, including publications from 2008 to 2023. The language was restricted to English, but there were no restrictions on the type of literature. The index format was Science Citation Index Expanded (SCI-EXPANDED), Current Chemical Reactions (CCR-EXPANDED), and Index Chemicus (IC).

### Data extraction and processing solution

**Figure 1:**
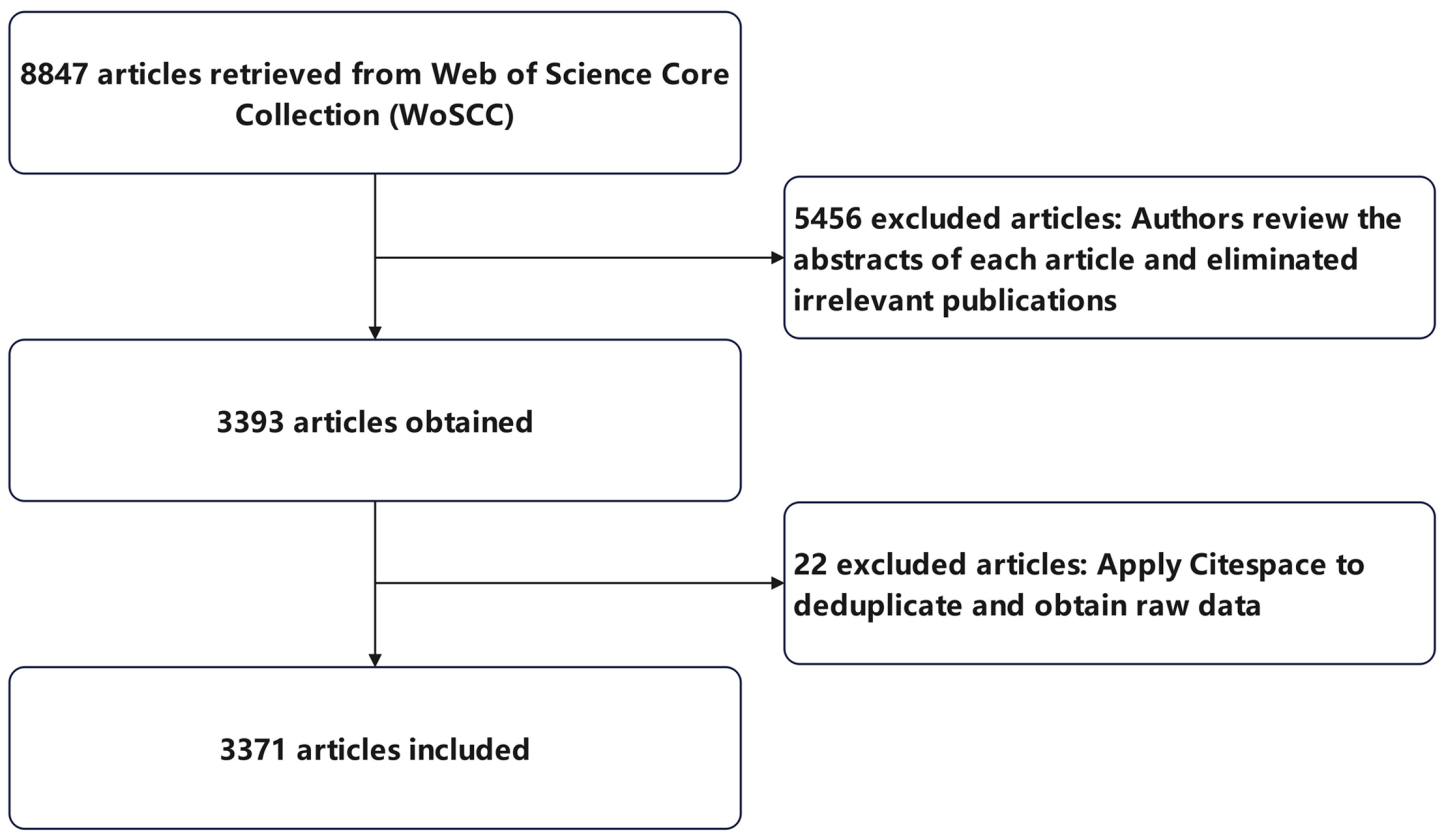
Flow chart of the study retrieval and selection process.

A total of 8,847 articles were retrieved. We manually screened the literature from each year and eliminated irrelevant publications. After exporting a plain text, 3,393 records that met the requirements were obtained. After deduplication, a total of 3,371 samples were obtained. A flow chart of the study retrieval and selection process is shown in Figure 1. These data do not involve identifiable information from patients and therefore the study did not require ethical review. We mainly used CiteSpace (v6.1, r6) for statistics, while VOSviewer 1.6.19 was used for analysis of journal publication volume and citation statistics.

### Software parameter settings

The CiteSpace settings were as follows: a “time slicing” value of 1 year was used; Pathfinder and Pruningsliced networks were selected for graph drawing; the factor K of the G-index was set to 25; and the TopN% was set to 10%. The image nodes and connections were adjusted according to the purpose of each analysis. Analyses using VOSviewer were performed with the default settings.

### Interpretation of main parameters

#### Node circle area and the link between nodes

The area of the node circle represents the number of papers published by each country/institution/author or the frequency of keywords in the co-occurrence network. Links represent the existence of cooperative or co-occurrence relationships, and the thickness of the connection represents the strength of the association.

#### Centrality

Centrality is an indicator that measures the importance of nodes in a network. The higher the centrality, the more links a given node has to other nodes.

#### Dual-map overlays

“Dual-map overlays” can be used to make the citation and citation relationships of various publications clearer (10). In this system, the distribution of cited journals is presented on the left, while the distribution of cited journals is presented on the right. The curve represents the citation line, which displays the specific citation relationship.

#### Cocitation analysis

Cocitation analysis was first proposed by the US central intelligence agency in 1973. Henry Smaller referred to the process of two articles being cited simultaneously by a third article as cocitation. This concept is now an important component of bibliometrics and has been proven to be a useful method for identifying key literature on interdisciplinary ideas (11).

#### Burst keywords

“Burst Keywords” are words that are frequently cited over a short period. Their distributions can predict frontier trends. In these visualizations, the red bar represents the popularity of the word during a period, while light green bars have the opposite meaning.

## Results

### Analysis of publication output

**Figure 2:**
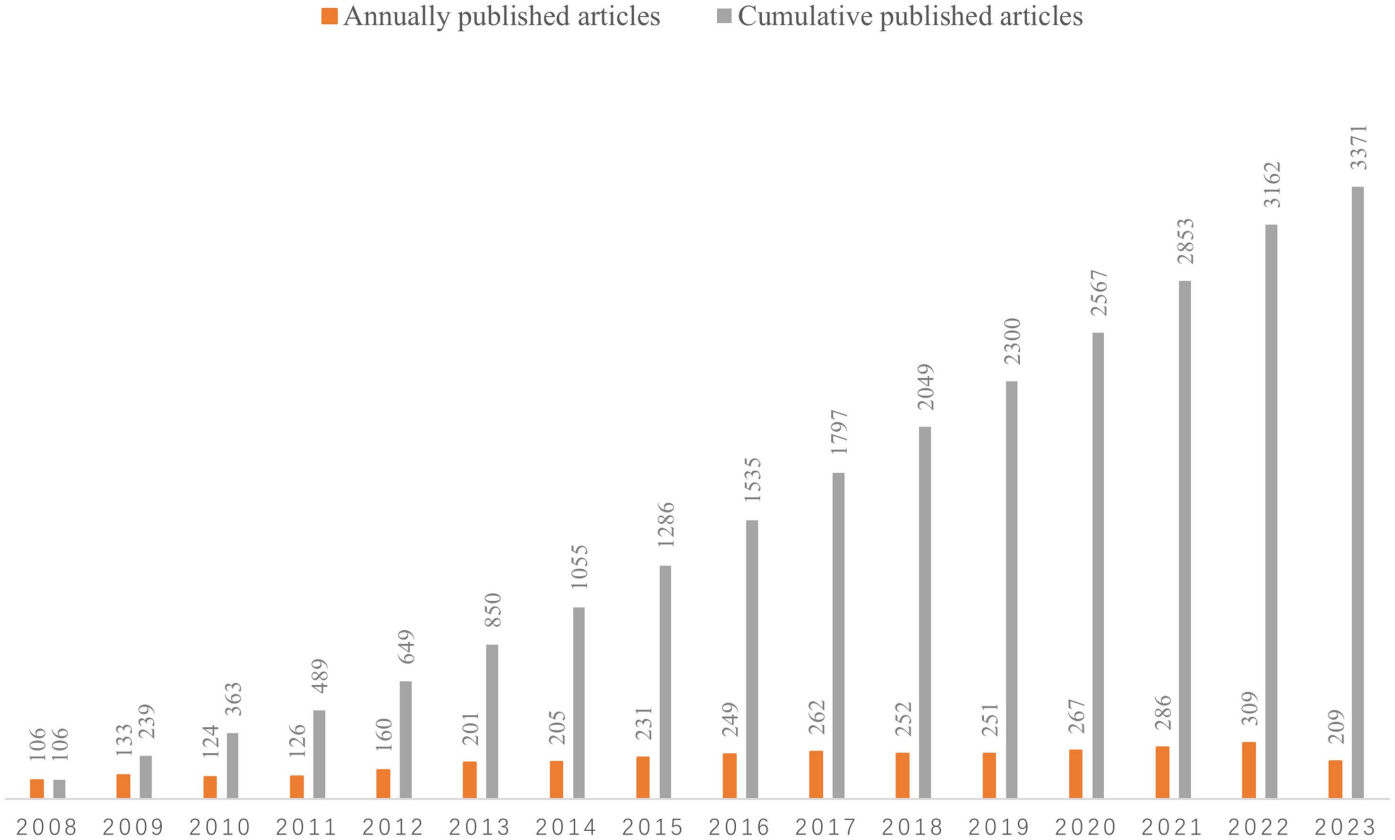
The number of annually published articles and cumulative number of published articles.

A total of 3,371 articles were included in this study. Figure 2 shows the number of published papers per year for 16 consecutive years, with the vertical axis representing the number of published papers and the horizontal axis representing the year. The popularity of brain atrophy research is gradually increasing, and the annual publication volume was stabled from 2016 to 2019. However, in 2020, the publication of papers entered an outbreak phase and was not impacted by the COVID-19 pandemic. Overall, brain atrophy is receiving increasing attention.

### Country of publication and international collaborative work analyses

**Figure 3:**
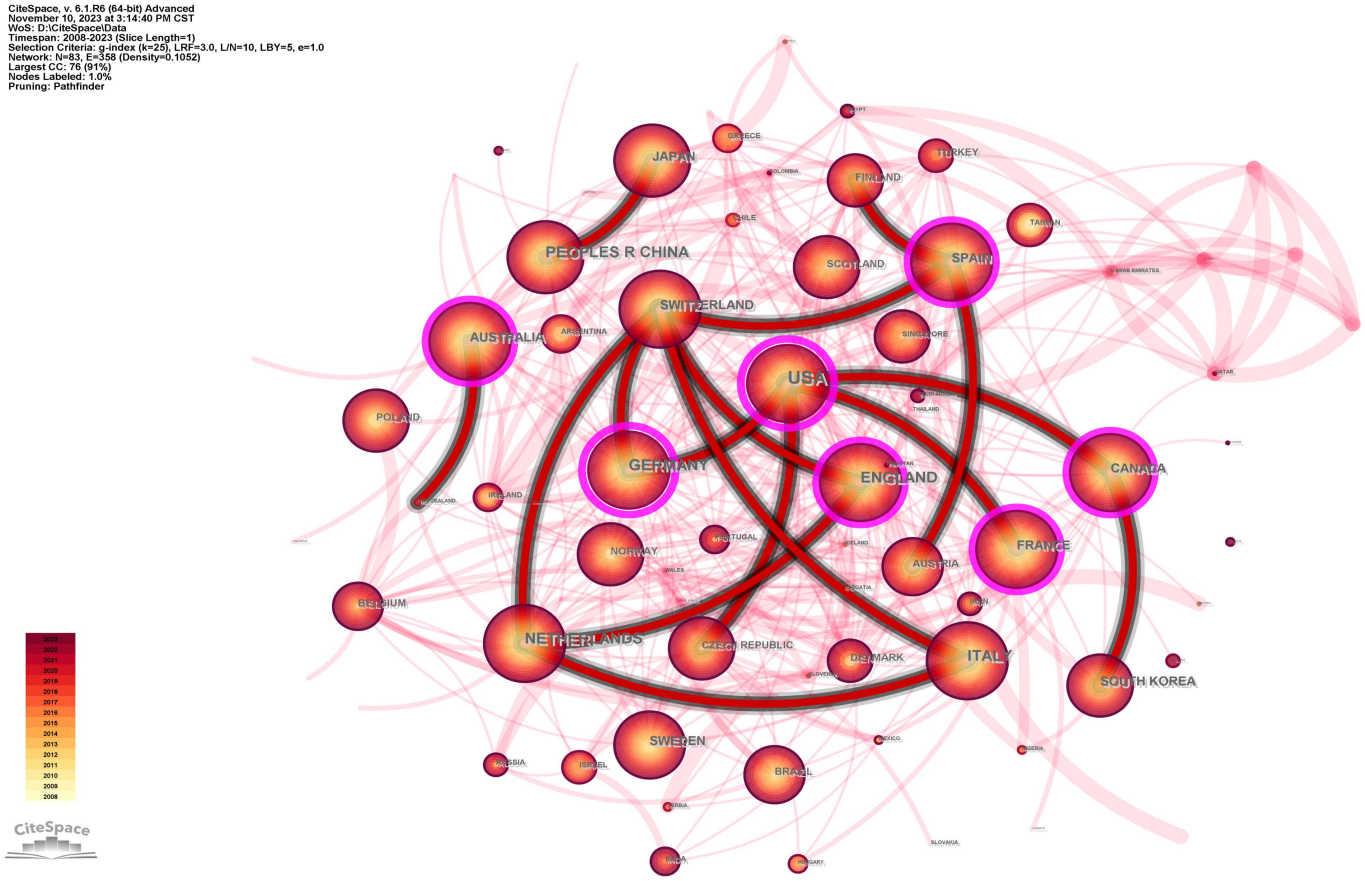
The collaboration network of countries/regions.

The included articles originated from 83 different countries. More than half of the articles came from the top three countries: the United States published the most articles (1,145, 34.0%), followed by England (409, 12.1%) and Italy (381, 11.3%), as shown in Table 1. Using CiteSpace and examining country nodes, Figure 3 shows a national collaborative network map. There are 83 nodes (N) and 358 connections (E) in the national cooperation relationship graph, with a network density of 0.1052, indicating that extensive scientific cooperation between countries has occurred. Among countries, the United States (0.23), Germany (0.21), and England (0.16) have higher centrality, indicating that they each play a crucial role in international cooperation. In addition, we found that Japan mainly cooperates with China and has the least cooperation with other countries, which may be related to its geopolitical environment.

**Table 1:** The top 10 countries in terms of publications.

Rank	Countries	Publications	Centrality
1	USA	1,145	0.23
2	England	409	0.16
3	Italy	381	0.03
4	Germany	319	0.21
5	Peoples’ Republic of China	310	0.05
6	The Netherlands	279	0.09
7	Canada	241	0.09
8	Japan	205	0.02
9	Australia	198	0.14
10	Spain	175	0.11

### Institution analysis

**Figure 4:**
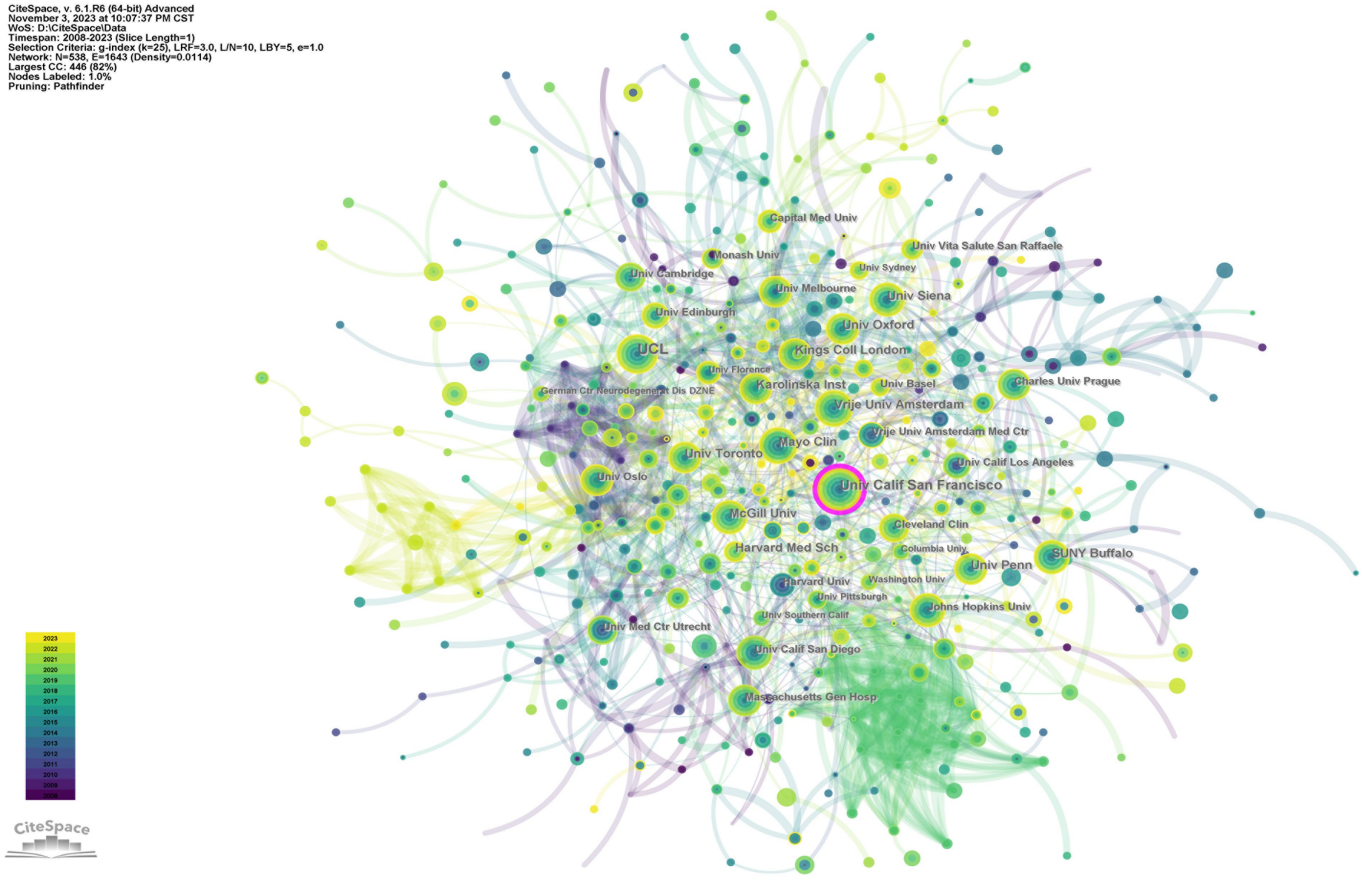
The collaboration network of institutions.

A total of 538 institutions published research articles analyzed in this study. Table 2 lists the 10 institutions with the highest number of publications. Among them, University College London was the top contributor (174, 5.2%), followed by the University of California, San Francisco (132, 4.0%) and University at Buffalo SUNY (90, 2.7%). Five of the top 10 institutions were from the United States. Using publishing institutions as nodes, a co-occurrence graph of publishing institutions was constructed (Figure 4). Only University of California, San Francisco (UCSF) with a centrality (0.13) greater than 0.1 can be considered a core center of interinstitutional cooperation. UCSF is among the most prestigious of the 23 branches of the California State University system. The UCSF Medical Center is part of this organization and has an excellent team of neurologists.

**Table 2:** The top 10 institutions in terms of publications.

Rank	Institutions	Publications	Centrality	Country
1	University College London	174	0.04	England
2	University of California, San Francisco	133	0.12	USA
3	University at Buffalo SUNY	90	0.06	USA
4	Mayo Clinic	87	0.06	USA
5	Vrije University Amsterdam	78	0.04	Netherlands
6	Harvard Medical School	73	0.01	USA
7	University of Toronto	70	0.04	Canada
8	McGill University	68	0.09	Canada
9	University of Pennsylvania	63	0.02	USA
10	Karolinska Institute	62	0.06	Sweden

### Journal analysis

Based on the VOSviewer analysis, there are a total of 589 journals that published relevant articles. Table 3 shows the top 10 journals. MULTIPLE SCLEROSIS JOURNAL has the most publications, followed by NEUROLOGY and then NEUROBIOLOGY OF AGING. Except for PLOS ONE, all the top 10 journals fell under the category of neurology. These journals all have a 2023 impact factor greater than 3, with NEUROLOGY having the highest (9.9), indicating the high quality of the literature on brain atrophy.

**Table 3:** The top 10 most productive journals.

Rank	Full journal title	Papers	Total citations	IF2023	WOS categories
1	MULTIPLE SCLEROSIS JOURNAL	289	2084	5.8	CLINICAL NEUROLOGY
2	NEUROLOGY	263	7,116	9.9	CLINICAL NEUROLOGY
3	NEUROBIOLOGY OF AGING	110	4,432	4.2	NEUROSCIENCES
4	JOURNAL OF ALZHEIMERS DISEASE	100	2059	4.0	NEUROSCIENCES
5	PLOS ONE	79	3,051	3.7	BIOLOGY
6	NEUROIMAGE-CLINICAL	79	1,583	4.2	NEUROIMAGING
7	JOURNAL OF NEUROLOGY	75	1,564	6.0	CLINICAL NEUROLOGY
8	EUROPEAN JOURNAL OF NEUROLOGY	72	945	5.1	CLINICAL NEUROLOGY
9	FRONTIERS IN AGING NEUROSCIENCE	69	843	4.8	NEUROSCIENCES
10	FRONTIERS IN NEUROLOGY	63	390	3.4	NEUROSCIENCES

**Figure 5:**
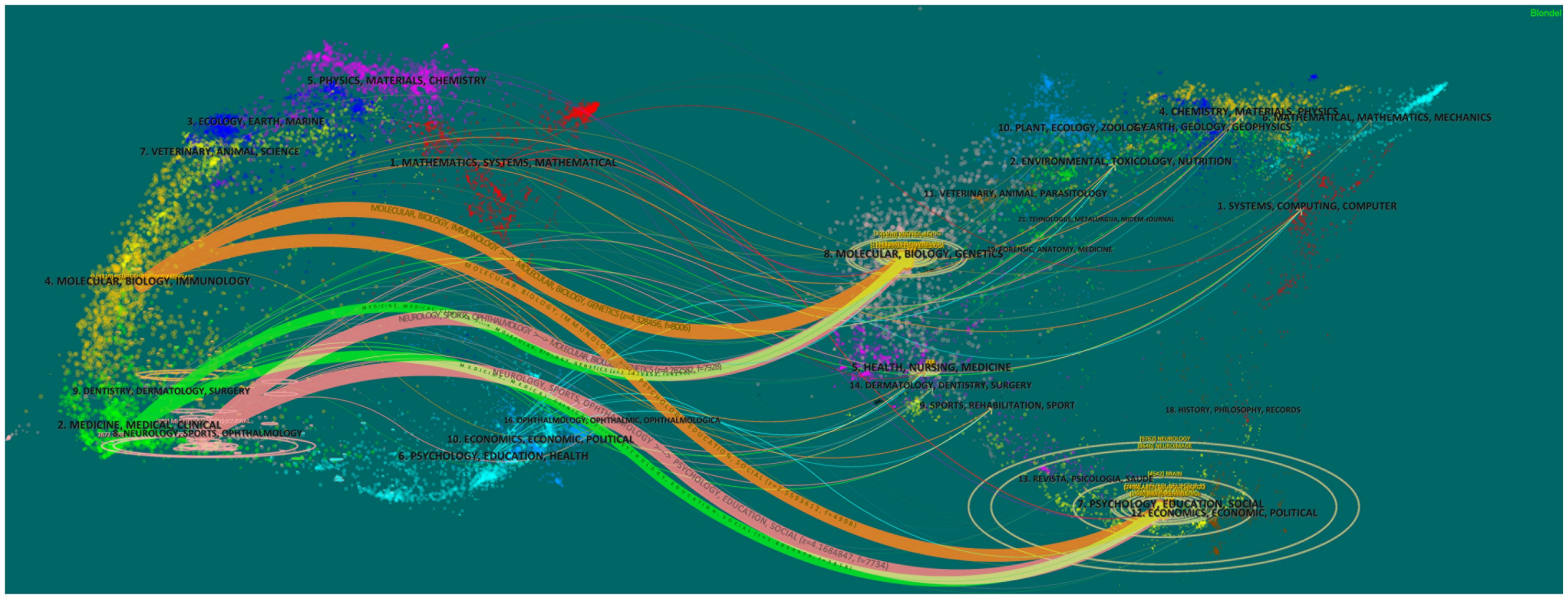
The dual-map overlay of brain atrophy.

In addition, we created a dual-map overlay (Figure 5). The journals that published cited articles on brain atrophy mainly come from the fields of Neurology, Sports, and Ophthalmology, while the journals with the most citations overal mainly come from the fields of Psychology, Education, Social, Economics, and Political.

### Analysis of contributing authors

**Figure 6:**
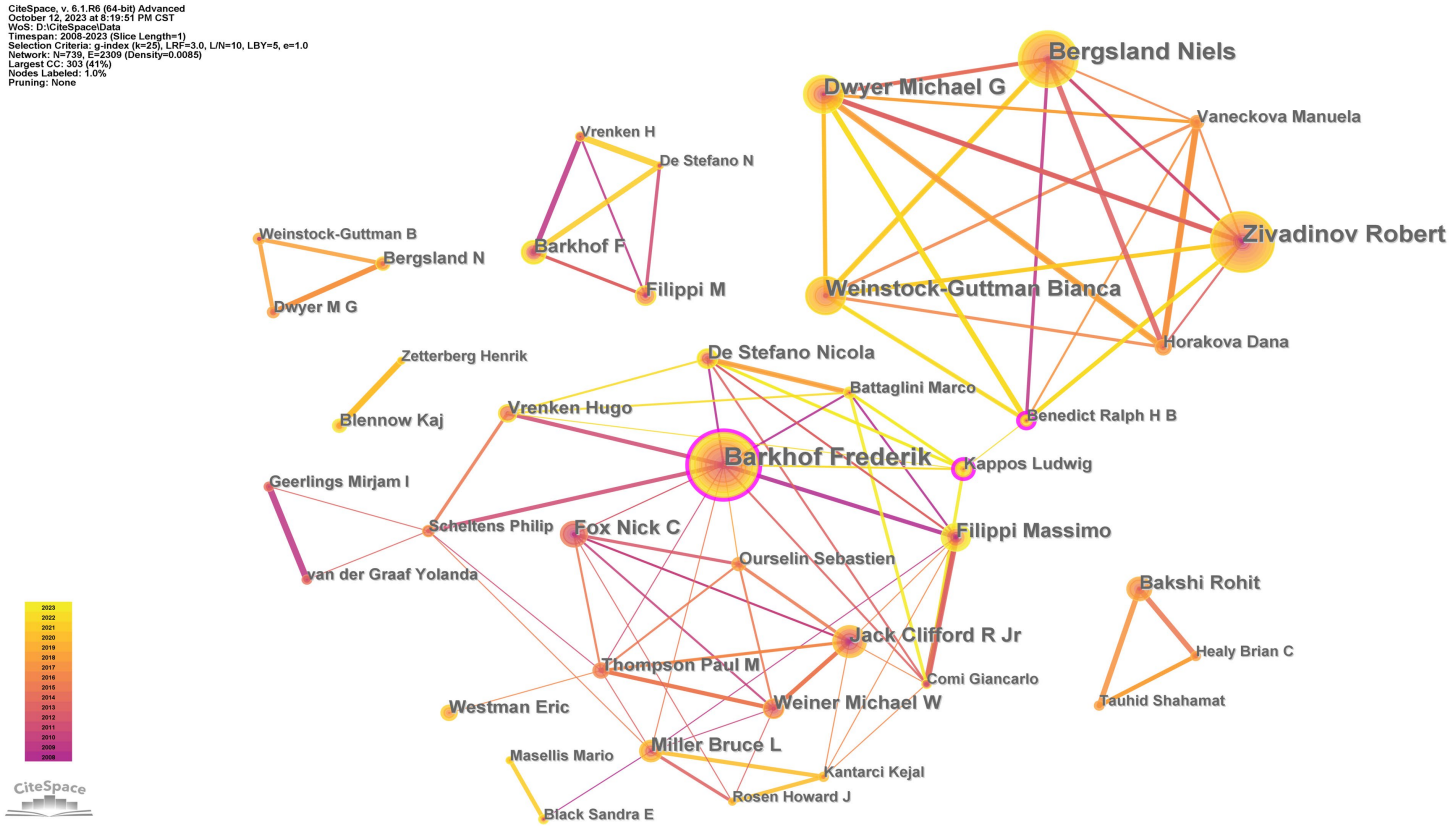
The collaboration network of authors.

A total of 645 authors contributed to the field of brain atrophy in the study period. Table 4 lists the 10 authors with the highest productivity. Barkhof Frederik is the most prolific author, having contributed 105 articles; the next most prolific authors are Zivadinov Robert (101) and Bergsland Niels (89). Using CiteSpace, an author collaboration network diagram was constructed with nodes representing authors (Figure 6). Core authors are the backbone of academic research in various disciplines. According to Price’s law, the minimum number of publications for a core author is *n* = 0.749 $\sqrt{N\max}$ , where Nmax is the number of papers published by the most prolific author (12). Therefore, the minimum number of articles to become the core author of brain atrophy research is 8, yielding a total of 79 core authors who have published a total of 1,568 articles, accounting for 46.5% all articles. Lotka’s Law requires that high-yield authors account for 50% of the total number of publications (13). Therefore, it can be considered that a stable core author group in the field of brain atrophy has almost formed.

**Table 4:** The top 10 most productive authors.

Rank	Authors	Papers
1	Barkhof Frederik	105
2	Zivadinov Robert	101
3	Bergsland Niels	89
4	Dwyer Michael G	73
5	Filippi Massimo	62
6	Weinstock-Guttman Bianca	56
7	Jack Clifford R	49
8	Fox Nick C	38
9	De Stefano Nicola	36
10	Vrenken Hugo	33

### Article cocitation analysis

Table 5 lists the ten papers with the highest cocitation strengths. According to the results of the CiteSpace analysis, the publication with the highest cocitation strength is “Deep gray matter volume loss drives disability worsening in multiple sclerosis” (14). It was written by Eshaghi Arman and published in ANNALS OF NEUROLOGY in 2018. This is a study from Europe aimed at investigating whether there is a spatiotemporal pattern of gray matter (GM) atrophy that is associated with faster disability accumulation in multiple sclerosis (MS). The results show that deep gray matter volume loss drives disability accumulation in MS and that temporal cortical GM shows accelerated atrophy in secondary progressive MS compared with relapsing–remitting MS.

**Table 5:** The top 10 papers with the highest cocitation strength.

Rank	Title	Authors	Publication year	Cocitation strength	Total citation
1	Deep gray matter volume loss drives disability worsening in multiple sclerosis	Eshaghi Arman	2018	58	247
2	Clinical relevance of brain volume measures in multiple sclerosis	De Stefano Nicola	2014	55	222
3	Within-subject template estimation for unbiased longitudinal image analysis	Martin Reuter	2012	54	1,525
4	Establishing pathological cut-offs of brain atrophy rates in multiple sclerosis	De Stefano Nicola	2016	53	197
5	NIA-AA Research Framework: Toward a biological definition of Alzheimer’s disease	Jack Clifford R Jr	2018	51	4,580
6	A Bayesian model of shape and appearance for subcortical brain segmentation	Patenaude Brian	2011	46	1,649
7	Diagnosis of multiple sclerosis: 2017 revisions of the McDonald criteria	Thompson Alan J	2018	39	3,604
8	Progression of regional gray matter atrophy in multiple sclerosis	Eshaghi Arman	2018	39	299
9	Brain MRI atrophy quantification in MS: from methods to clinical application	Rocca Maria A	2017	39	156
10	Hypothetical model of dynamic biomarkers of the Alzheimer’s pathological cascade	Jack Clifford R Jr	2010	38	3,206

### Keyword analysis

**Figure 7:**
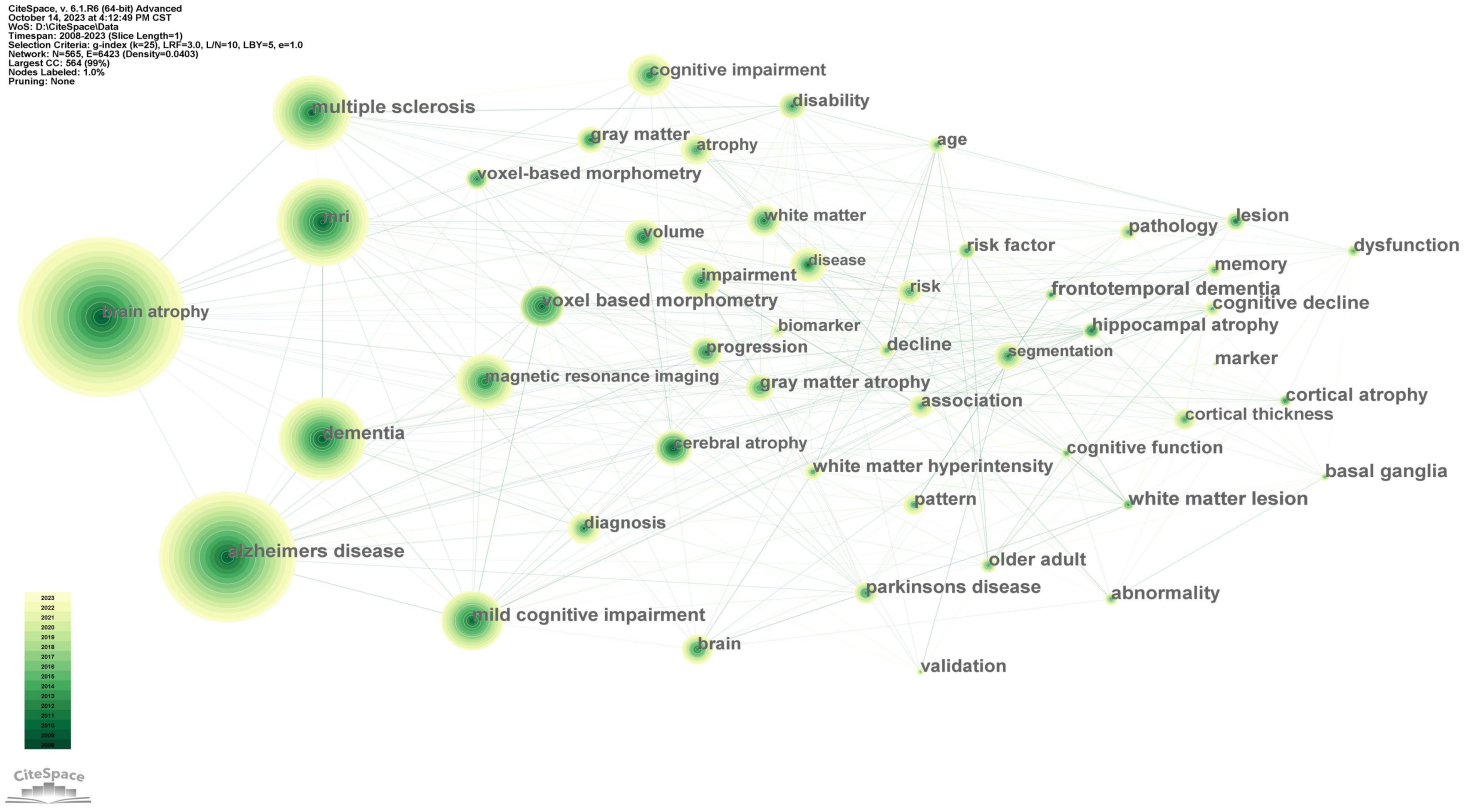
Map of keywords.

Key words are a reflection of an article’s topics. If a keyword appears repeatedly, Its representative topic is popular research. Table 6 lists the top 10 most frequently occurring keywords in the included. The most common was “brain atrophy” (900), followed by “Alzheimer’s disease” (740) and “MRI” (506). In addition, using CiteSpace, a keyword co-occurrence network graph Was constructed with nodes representing keywords, as shown in Figure 7.

**Table 6:** The top 10 keywords with the highest frequency.

Rank	Keywords	Frequency
1	Brain atrophy	900
2	Alzheimer’s disease	740
3	MRI	506
4	Dementia	471
5	Multiple sclerosis	422
6	Mild cognitive impairment	344
7	Magnetic resonance imaging	326
8	Voxel based morphometry	255
9	Cognitive impairment	252
10	Volume	221

**Figure 8:**
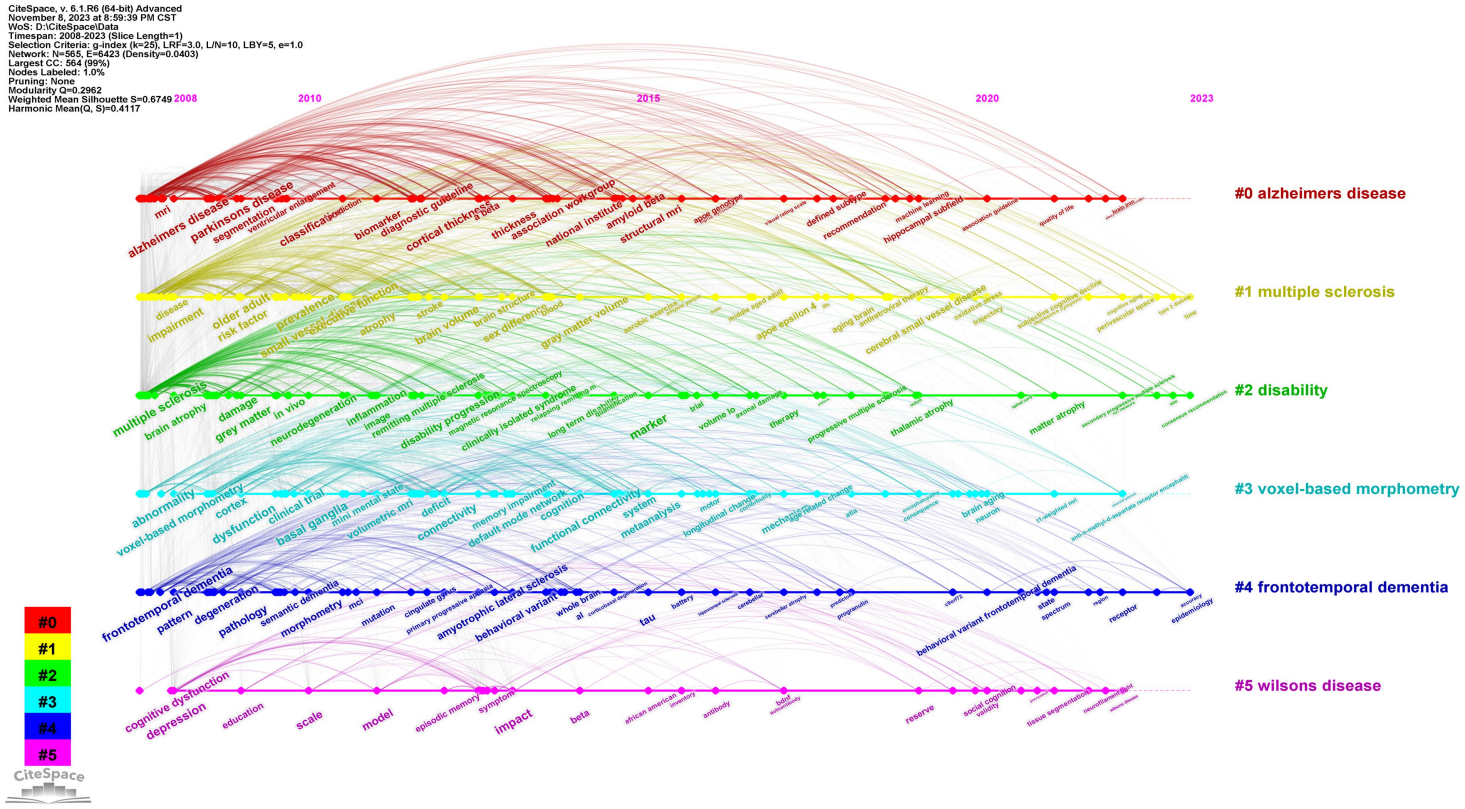
Timeline view of keyword clustering analysis.

We use CiteSpace to generate a timeline view of keyword clustering (Figure 8). That the specific clusters include “Alzheimer’s disease,” “multiple sclerosis,” “disability,” “voxel-based morphometry,” “frontotemporal dementia,” and “Wilsons disease” with sizes of 116, 115, 111, 97, 81, and 44, respectively. These labels reflect the main classifications in the field of brain atrophy research.

**Figure 9:**
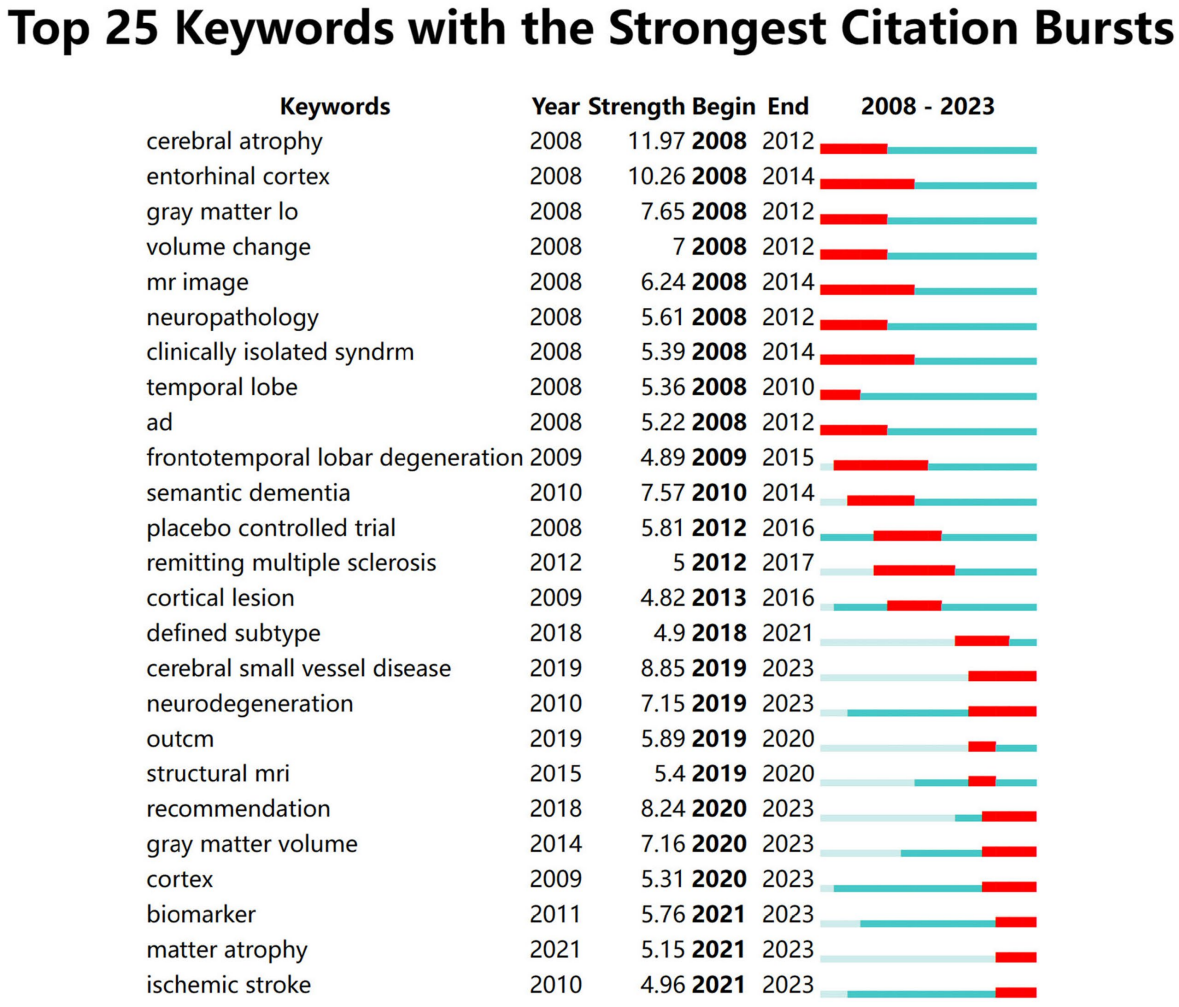
The top 25 keywords with the strongest citation bursts.

Figure 9 shows the top 25 keywords with the strongest citation bursts. “cerebral small vessel disease,” “neurodegeneration,” and “cortex/gray matter volume” may become hot research topics in the coming years.

## Discussion

### General information

A total of 3,371 publications were included in the analysis. From 2008 to 2023, the number of publications increased annually. This indicates that research on brain atrophy is increasingly being valued. From an international perspective, the United States has the highest research output (1,145, 34.0%) and the highest centrality (0.23). Institutional analysis showed that University College London had the highest number of publications (174, 5.2%). The University of California, San Francisco (UCSF) had the highest centrality (0.12), indicating that it is the core of international cooperation.

In terms of authors, Barkhof Frederik was the most prolific author (105), followed by Zivadinov Robert (101); these individuals can be considered leaders in this field. A search from Web of Science showed that Barkhof Frederik (1,715 total documents, 146 of H-index) works at the Amsterdam UMC and has made significant achievements in treating Alzheimer’s disease. He has received the prestigious recognition of being a highly cited researcher in the field of neuroscience and behavior for eight consecutive years (2016–2023). In addition, we found that Zivadinov Robert (742 total documents, 68 of H-index) from Buffalo SUNY has stopped publishing articles since 2020, which is a regrettable fact. Classified according to Price’s Law, 79 core authors published 1,568 articles. This accounts for 46.5% of the total number of publications. Therefore, a stable group of core authors in the field of brain atrophy will imminently form.

**Figure 10:**
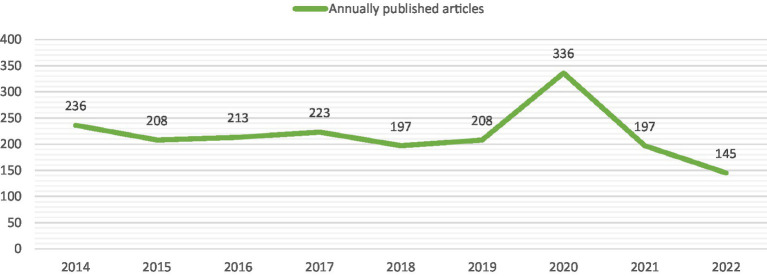
The number of annually published articles of MULTIPLE SCLEROSIS JOURNAL.

All the included papers were published by one of 589 journals. Most of the top 10 journals are focused in the field of neurology and are high quality. MULTIPLE SCLEROSIS JOURNAL has the most publications. It comes from the UK and focuses on fields of multiple sclerosis, neuromyelitis optica and other central nervous system autoimmune diseases. A graph was created to show the annual publication count of this journal from 2014 to 2022 (Figure 10). We found that the number of publications decreased rapidly in the past 2 years, which may affect its leading position in the field of brain atrophy in the future. From the dual-map overlay, the journals with the most cited publications on brain atrophy mainly come from the fields of Neurology, Sports, and Ophthalmology, while the journals with the most cited publications in general mainly come from the fields of Psychology, Education, Social, Economics, and Political.

### Hotspots and frontiers

To evaluate the current research hotspots and frontiers, we used CiteSpace for keyword analysis. The results showed that from 2008 to 2023, the most frequently occurring keyword was brain atrophy (900), followed by Alzheimer’s disease (740) and MRI (506). We also found that “Alzheimer’s disease” and “multiple sclerosis” are currently the two most popular keyword clusters.

Alzheimer’s disease (AD) is a major global health challenge. According to incomplete statistics, there are over 25 million dementia patients in the world today, most of whom have AD (15). Research has shown that these patients are mostly concentrated in low-income and middle-income countries, thus representing a heavy economic burden (16). Brain atrophy is a major characteristic manifestation of this disease. In recent years, the evaluation of brain atrophy neuromarkers based on MRI has been proven to be effective for the diagnosis of Alzheimer’s disease (17, 18). Among the various subtypes of brain atrophy, hippocampal atrophy may be the best AD marker (19). Low hippocampal volume has been recognized by the European Medical Agency (EMA) for clinical trials of Alzheimer’s disease in the mild cognitive impairment (MCI) stage (20). The development of new automatic/manual segmentation methods is a current research hotspot aimed at providing faster and more accurate quantitative measurements of hippocampal volume (21). This may pave the way for further application of brain atrophy in the field of AD.

MS is the most common demyelinating disease, with a higher incidence rate in high-income countries (22). Brain atrophy has been recognized as an important feature of late-stage MS. The loss of brain tissue volume begins in the early stages of disease and is associated with the degree of physical and cognitive disability (4, 23). Some studies suggest that brain atrophy is the best predictor of future disability in MS patients (24, 25). With the introduction of new advanced imaging technologies, understanding of MS will increase (26). In addition, reducing the rate of brain atrophy has gradually increased in popularity as a key endpoint in clinical studies (27). Perhaps in the future, brain atrophy will bring new hope for the treatment of MS.

According to the analysis of burst keywords, “cerebral small vessel disease,” “neurodegeneration,” and “cortex/gray matter volume” may become hot research topics in the coming years.

### Cerebral small vessel disease

Cerebral small vessel disease (SVD) is the main cause of vascular dementia (28). Compared to normal elderly individuals, patients with hereditary SVD have a higher rate of brain atrophy (29, 30). Long-term chronic ischemia of cerebral blood vessels is the core mechanism underlying brain atrophy (31–33). One study showed that the brain volume of SVD patients decreases over time with highly sensitive measurements (34). This can serve as an alternative indicator for monitoring disease progression and evaluating the effectiveness of treatment interventions. SVD exhibits heterogeneous etiologies. The most common forms are arteriosclerosis and cerebral amyloid angiopathy (35). However, there is currently limited research on the association between different types of SVD and brain atrophy (36). This may be the main future SVD research direction.

### Neurodegeneration

The appearance of brain atrophy on MRI is now regarded as a biomarker for neurodegeneration (37). As a crucial pathological link, neurodegeneration occurs in various diseases such as AD. AD is defined pathologically as amyloid plaques and tau neuronal tangles (38). However, the role of β-amyloid protein (Aβ) in sedimentation has been controversial for decades. Some studies suggest that Aβ in AD is related to neurodegeneration and brain atrophy (39, 40). Another study indicated that the Aβ load is not related to the rate of brain atrophy (41). Discussions about this aspect may continue to be important in the future.

### Cortex/gray matter volume

Researchers have been paying increasing attention to the measurement of GM volume in recent years. Taking MS as an example, white matter (WM) plaques are recognized as the cause of brain atrophy. However, some studies have pointed out a lack of correlation between these factors (42–44). Damage to the GM may be an important cause of MS (27). Indeed, due to the immaturity of gray matter myelin sheath detection technology, the importance of GM injury in MS pathology has long been overlooked (45). Until recent years, research on the measurement of gray matter volume has gradually increased and may become a new research hotspot.

### Strengths and limitations

This study represents a groundbreaking visual analysis of the field of brain atrophy based on bibliometrics, facilitating understanding of the current research status and future hot trends. The joint application of CiteSpace and VOSviewer makes the results more comprehensive and accurate. However, the manual screening of the literature that was performed may have resulted in missing information. Moreover, the absence of some synonyms leads to the omission of some documents in the search process. In addition, due to software limitations, only the WoSCC database could be used. If conditions allow in the future, it is necessary to introduce more databases in later studies.

## Conclusion

We have summarized 3,371 articles in the field of brain atrophy published from 2008 to 2023 to determine the current state and future trends of this research topic. The results indicate that the attention in this field is gradually increasing, that extensive international cooperation already exists, and that the quality of articles is generally high. In addition, a stable core author group has almost formed.

## Data availability statement

The original contributions presented in the study are included in the article/supplementary material, further inquiries can be directed to the corresponding authors.

## Author contributions

JW: Data curation, Writing – original draft. TC: Data curation, Writing – review & editing. JX: Writing – review & editing. SZ: Writing – review & editing. YJ: Writing – review & editing. HZ: Conceptualization, Funding acquisition, Writing – review & editing. WZ: Conceptualization, Writing – review & editing.
